# Characteristics of Cancers in Community Members Exposed to the World Trade Center Disaster at a Young Age

**DOI:** 10.3390/ijerph192215163

**Published:** 2022-11-17

**Authors:** Rebecca Lynn Florsheim, Qiao Zhang, Nedim Durmus, Yian Zhang, Sultan Pehlivan, Alan A. Arslan, Yongzhao Shao, Joan Reibman

**Affiliations:** 1 Department of Medicine, NYU Grossman School of Medicine, New York University, New York, NY 10016, USA; 2 Department of Population Health, NYU Grossman School of Medicine, New York University, New York, NY 10016, USA; 3 Department of Pathology, Johns Hopkins School of Medicine, Baltimore, MD 21205, USA; 4 Department of Obstetrics and Gynecology, NYU Grossman School of Medicine, New York University, New York, NY 10016, USA

**Keywords:** World Trade Center, WTC survivors, environmental exposure, cancer, cancer characteristics, young adult, children

## Abstract

The destruction of the World Trade Center (WTC) towers on 11 September 2001 (9/11) released tons of dust and smoke into the atmosphere, exposing hundreds of thousands of community members (survivors) and responders to carcinogens. The WTC Environmental Health Center (WTC EHC) is a federally designated surveillance and treatment program for community members who were present in the New York City disaster area on 9/11 or during the months that followed. WTC EHC enrollment requires exposure to the WTC dust and fumes and a federally certifiable medical condition, which includes most solid and blood cancers. Several studies have described the prevalence and characteristics of cancers in responders and survivors exposed to the WTC dust and fumes as adults. Cancers in those exposed at a young age warrant specific investigation since environmental toxin exposure at a younger age may change cancer risk. We describe the characteristics of 269 cancer patients with 278 cancer diagnoses among WTC EHC enrollees who were young in age (aged 0 to 30) on 9/11. These include 215 patients with a solid tumor (79.9%) and 54 with a lymphoid and/or hematopoietic cancer (20.1%). Among them, 9 patients had a known second primary cancer. A total of 23 different types of cancer were identified, including cancer types rare for this age group. Many were diagnosed in individuals lacking traditional cancer-specific risk factors such as tobacco use. The current study is the first to report specifically on cancer characteristics of younger enrollees in the WTC EHC program.

## 1. Introduction

The destruction of the World Trade Center (WTC) towers on 11 September 2001 (9/11) released millions of tons of aerosolized dust and smoke into the atmosphere, exposing responders and the surrounding community [1,2]. Community members, including those who lived, worked, cleaned contaminated sites, were passing through, or attended schools in the disaster area (survivors) had potential for adverse health effects from acute exposures to the dust generated by the collapsing towers on 9/11, as well as from chronic exposures to resuspended dust, or fumes from the fires that continued to burn through December 2001 [3,4,5,6,7]. An estimated 25,000 children aged 18 years or younger were living or attending school in the area close to the WTC site on or after 9/11 [8], including 8000 grade school (K-12) students who were present in the disaster area on 9/11 [9]. Younger adults, aged 18–30, were also present in the disaster area after 9/11 as residents, students, or local workers. Many residents on the west side of lower Manhattan, including those with children, were temporarily evacuated; those on the east side remained in their homes, some without water or electricity. Many school children witnessed the destruction on 9/11 and were evacuated from their educational institutions emergently. Local schools were temporarily closed, with subsequent displacement of affected children.

Indoor and outdoor dust and fumes included respirable particulate matter including PM10 and PM2.5, as a combination of pulverized cement, glass fibers, lead, asbestos, and combustion products. They also included complex mixtures of volatile chemicals such as benzenes, polycyclic aromatic hydrocarbons, asbestos, polychlorinated biphenyls, and polychlorinated furans and dioxins [2,3,10,11,12,13,14,15,16], all of which are potential carcinogens.

In the years following 9/11, through the concerted efforts of unions, community-based organizations, and academic centers, programs were developed to monitor and treat illness in responders and community members. With the passage of the James Zadroga 9/11 Health and Compensation Act H.R. 847 in 2010 (Zadroga Act), these programs were combined into the WTC Health Program (WTCHP) under the Centers for Disease Control and Prevention (CDC), National Institutes of Safety and Occupational Health (NIOSH) [3]. The original Clinical Centers of Excellence within the WTCHP included four groups serving responders, and one serving community members (survivors) at the New York City Health and Hospitals WTC Environmental Health Center (WTC EHC) [3,17,18]. The WTC EHC included an adult and pediatric program open to all age groups. A concerted recruitment effort to include children involved collaboration with numerous community-based organizations, local pediatricians, parent-teacher associations, tenant’s organizations, and the NYC Department of Health and Hygiene WTC Health Registry (WTCHR), as well as multi-media campaigns. 

Several studies have now described the prevalence of cancer in WTC-exposed populations with a focus on responders [19,20,21,22,23,24,25,26,27,28]. Cancers in survivors have been described in the WTCHR, and we described cancer characteristics in the WTC EHC population [29,30,31,32]. The exposure to 9/11 dust and fumes at a very young age has been associated with adverse health outcomes [33,34,35,36,37]. Children have potential for relatively higher environmental contaminant exposure than adults due to differences in their physiologic function, increased surface-to-volume ratio, and different behaviors compared with adults, including increased hand-to-mouth behaviors [38]. Studies in the general population also indicate in utero exposures to environmental toxins may influence cancer risk by modifying biological pathways associated with carcinogenesis and that exposure in younger individuals may increase cancer risk by increasing overall lifetime exposures or by impacting critical developmental periods [39]. Thus, those exposed to the 9/11 toxins at a young age may have different cancer characteristics compared with those exposed as older adults. To date, there has been no formal report describing the characteristics of cancer diagnoses in WTC-exposed individuals who were exposed at young age (EYA), namely, as children, adolescents, or adults aged 18–30 on 9/11. To fill this knowledge gap, we report the characteristics of the cancers in EYA enrollees in the WTC EHC as of 31 July 2021.

## 2. Materials and Methods

### 2.1. Patient Enrollment

The WTC EHC was established in response to requests from the community in the years following 9/11, and was later designated by CDC/NIOSH as the Center of Excellence in the WTCHP for community members, called “Survivors” under the Zadroga Act [3,4,30]. Patients self-refer to the WTC EHC, and enrollment requires defined WTC exposure and at least one “certifiable condition,” which may include an aerodigestive disorder, mental health symptoms consistent with PTSD, depression or anxiety, or a cancer diagnosis [3,18]. Individuals born within 9 months of 9/11 are eligible for enrollment in the WTC EHC. Once enrolled in the WTC EHC, patients are recalled for ongoing monitoring with repeated standardized medical and mental health evaluations. Demographic characteristics were obtained from questionnaires completed upon enrollment in the WTC EHC, as previously described [4,30].

### 2.2. Exposure Assessment 

Survivors have potential complex exposure to the indoor and outdoor WTC dust and fumes. Community members may have had acute exposure to the dust and fumes on 9/11, including exposure to the massive dust clouds that resulted from the collapse of the WTC towers (dust cloud yes/no). Survivors may also have had chronic exposure to resuspended dust as residents, students, volunteers, commuters, or as local workers, most of whom returned to work approximately one week after the disaster. Children had potential for exposure as local residents, or as students in the 51 nurseries, preschools and primary and secondary schools within the disaster area on 9/11 [33,35,40,41,42]. All WTC EHC enrollees (or their parents) completed exposure questionnaires identifying their specific exposures to WTC dust and fumes. 

### 2.3. Cancer Information 

Cancer diagnoses data were obtained by patient reports or from the New York State (NYS) Tumor Registry. Data were maintained in the WTC EHC pan-cancer database (WTC EHC PCDB) [29,30]. The WTC EHC PCDB includes “certifiable” cancers as defined by WTCHP guidelines reflecting CDC Minimum Latency requirements for specific cancers, as well as uncertified cancers diagnosed after 9/11 that may not meet minimum latency requirements. All cancer diagnoses were confirmed by a review of the pathology or cytology reports and medical records, or from data obtained from the NYS Tumor Registry. Only primary cancers were included in this report.

The date of the first diagnostic pathology or cytology report was documented as the date of cancer diagnosis. This date was also used to calculate the age at diagnosis. Cancer characteristics were derived from clinical and pathology reports, review of medical records, and state tumor registries [29,30]. We included patients enrolled as of 31 July 2021 in this study. 

### 2.4. Inclusion Criteria

For this analysis, we included patients who were enrolled in the WTC EHC between May 2002 and 31 July 2021 and who were ≤30 years old on 9/11 and had a cancer diagnosis. Because non-melanoma skin cancers are very common, often under-diagnosed, and commonly treated within primary care and, therefore, likely to be under-reported to the state cancer registries, they are often excluded from the reporting of cancer statistics. Taking this into account, we decided to exclude non-melanoma skin cancers from this report. This research study was approved by the New York University School of Medicine Institutional Review Board (IRB number: i06-1). Cancer patients with IRB approval were analyzed after personal identifiers were removed to review de-identified data (IRB number: i06-1_MOD49). 

### 2.5. Statistical Analysis

Descriptive statistics were generated to summarize population characteristics, including medians and ranges to represent continuous variable data and counts and percentages to represent binary or categorical variable data. WTC exposure status and demographic characteristics were numerically summarized for the cancer group. Bar graphs were used to display distributions (frequencies) of various cancer diagnoses and in strata based on age at 9/11 or gender. We utilized statistical software RStudio version 3.6.3 to conduct these analyses.

## 3. Results

### 3.1. Participants

**Figure 1:**
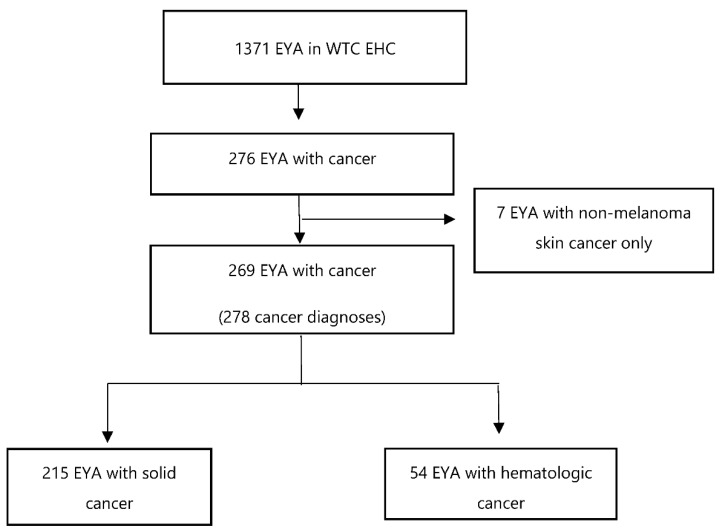
Distribution of cancers in WTC survivors who were exposed at a young age (EYA) enrolled in the WTC EHC as of 31 July 2021.

We identified 1371 EYA patients in the WTC EHC (Figure 1). Among them, 276 patients (20.1%) had a diagnosis of any cancer type. After excluding non-melanoma skin cancer cases (n = 7), we included 269 EYA with 278 cancer diagnoses. Of the patients with a cancer diagnosis, most patients (n = 215, 79.9%) had a solid cancer, with fewer (n = 54, 20.1%) having a lymphoid or hematopoietic tissue cancer diagnosis. 

Some survivors had missing data on various individual characteristics. The available data on each of characteristics of EYA enrollees with variable total sample size are summarized and shown in Table 1. The median age on 9/11 was 26, with a range of 0 to 30 years. The majority of EYA members with cancer (63.9%) were women, and there was a diverse distribution of race and ethnicity. Most (65.7%) had an elevated body mass index (BMI). We characterized members by their potential for exposure and 141 (54.2%) were local workers, 52 (20.0%) were residents, 49 (18.8%) were students, and 18 (7.0%) were others. Approximately half (45.8%) reported having been caught in the WTC dust cloud on 9/11. Most EYA members with cancer (86.6%) were never smokers. We also stratified the characteristics of the patients who were very young (age 0–10), adolescent (age 11–20) or young adults (age 21–30) on 9/11. Among the six cancers diagnosed in individuals aged 10 or younger on 9/11, two were papillary thyroid cancers, two were Hodgkin’s lymphoma, one was a Wilms’ kidney cancer, and one was acute lymphoblastic leukemia. 

### 3.2. Cancer Types

**Figure 2:**
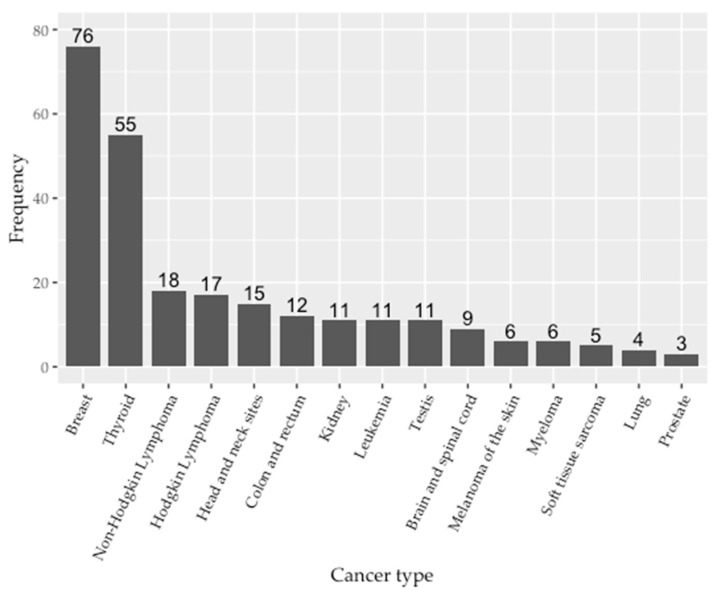
Top 15 cancer diagnoses in EYA included in the WTC EHC cancer analysis.

Within the 269 cancer patients, 23 distinct cancer sites were identified with 278 cancer diagnoses, including 9 patients (3.3%) with a known second primary cancer. The fifteen most common cancer diagnoses in EYA are shown in Figure 2. Overall, breast (n = 76, 27.3%) and thyroid (n = 55, 19.8%) cancer diagnoses were most common, followed by lymphoma (n = 35, 12.6%) and cancer of the head and neck sites (n = 15, 5.4%). As referenced above, the 11 cases of kidney cancer included one EYA diagnosed with Wilms’ tumor at age of 7. One rare cancer case not included in this figure was that of a peritoneal mesothelioma. 

**Figure 3:**
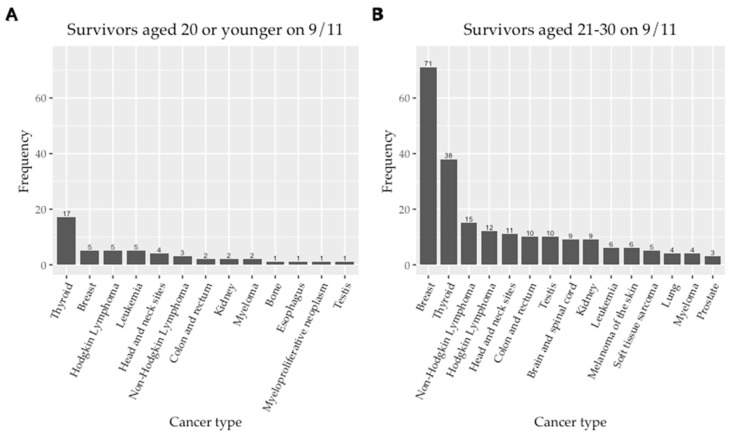
(**A**) Frequency of all cancer diagnoses (total 13 types) in EYA aged 20 or younger on 9/11; (**B**) Frequency of top fifteen cancer diagnoses in EYA aged 21–30 on 9/11 included in the WTC EHC cancer analysis.

From Table 1, we note that there are only six EYAs in the 0–10 age category. Among them, two were diagnosed with thyroid cancer, two with lymphoma, one with leukemia and one with kidney cancer. We plotted the distribution of the top 15 cancer diagnoses in EYA by those age 20 or younger and those aged 21–30 on 9/11 (Figure 3). Only thirteen cancer sites were identified in the younger age group. The vast majority (81.2%) of cases represented in Figure 3 were identified in the group that was older on 9/11.

**Figure 4:**
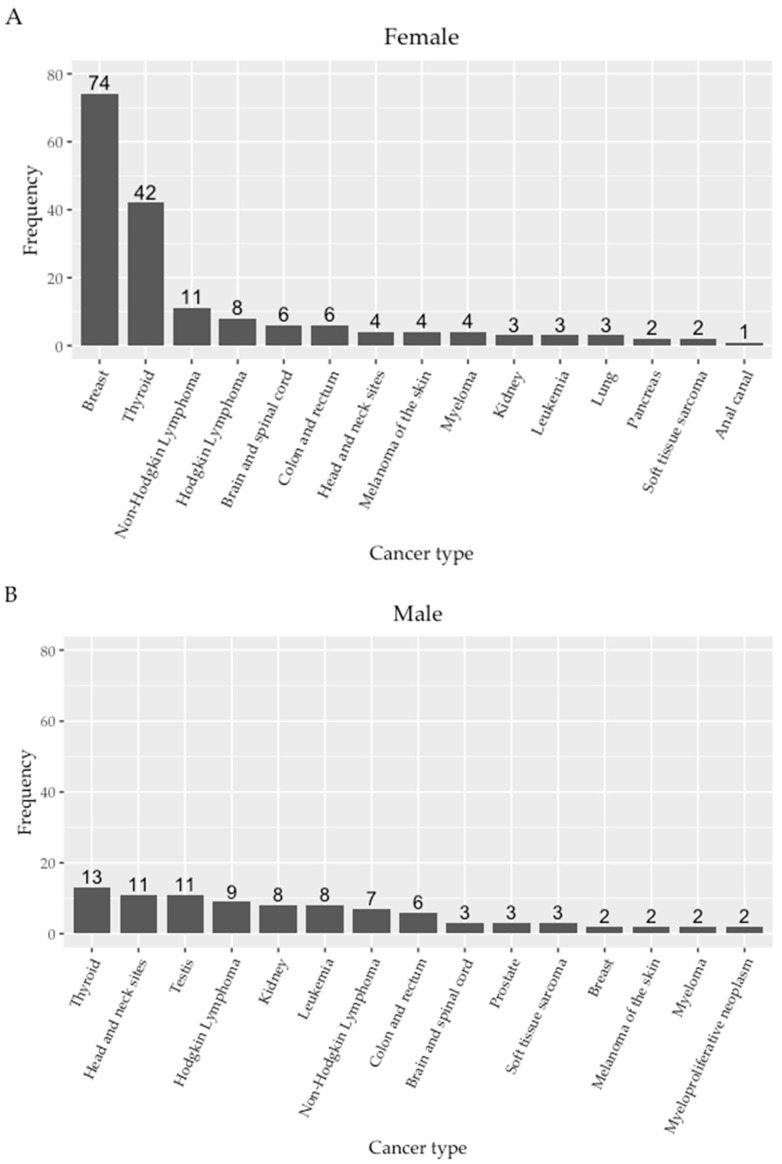
Frequency of top fifteen cancer diagnoses in female (**A**) and male (**B**) EYA included in the WTC EHC cancer analysis.

The frequency distribution of the cancers differed between male and female EYA (Figure 4). Among EYA women diagnosed with cancer, breast cancer was the most common cancer diagnosis (42.8% of cancers in women), followed by thyroid (24.2%), non-Hodgkin’s lymphoma (6.4%), Hodgkin’s lymphoma (4.6%), and cancer of the brain and spinal cord (3.5%). In EYA men, the thyroid was the most common cancer site (14.4%), followed by the head and neck (12.2%) and testis (12.2%), and Hodgkin’s lymphoma was also common (10.0%). Kidney cancers were more represented in male EYA (n = 8) compared with female (n = 3). There were two breast cancer cases identified in male EYA. 

The characteristics of EYA diagnosed with each of the top 10 solid cancers are shown in Table 2. Of the 204 solid cancer cases, the median age at cancer diagnosis was 38 (range 7–48 years). Breast cancer was diagnosed at a median age of 40 (range 27–48 years), and thyroid cancer at a median age of 35, with the youngest individual diagnosed at age 13. All the lung cancers (n = 4) were non-small cell lung cancers, including three adenocarcinoma and one carcinoid cancer subtypes. Because certain cancers in the general population are typically associated with a smoking history or elevated BMI, we examined the association of several cancer types with these risk factors among EYAs at WTC EHC. Lung, head and neck, colorectal and kidney cancers are frequently associated with a history of tobacco use in the general population [43]. In contrast, most EYA diagnosed with head and neck (11 out of 13, 84.6%), colorectal (8 out 10, 80.0%), and kidney (8 out of 10, 80.0%) cancers were never smokers. In general, elevated BMI has been associated with cancer diagnoses, including colorectal cancer, kidney cancer, thyroid cancer, and head and neck cancers [44]. Similarly, most EYA diagnosed with colorectal (n = 4 out of 7, 57.1%), kidney (n = 7 out of 10, 70.0%), thyroid (n = 21 out of 33, 63.6%), and head and neck (n = 4 out of 9, 44.4%) cancers had an elevated BMI.

### 3.3. Hematologic Malignancies

**Figure 5:**
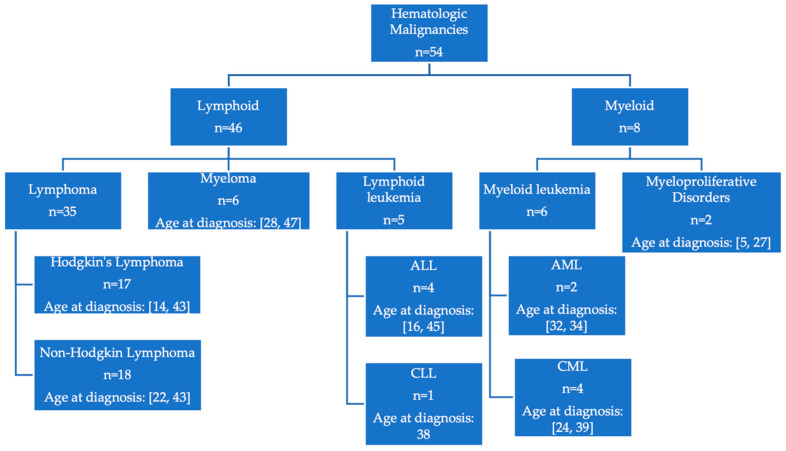
Distribution and range of age at diagnosis of hematologic malignancies in EYA. ALL = Acute lymphoblastic leukemia; AML = Acute myeloid leukemia; CLL = Chronic lymphocytic leukemia; CML = Chronic myelogenous leukemia.

Subtypes of hematologic malignancies diagnosed in EYA are shown in Figure 5. Of the 54 reported malignancies, the majority (n = 46, 85.2%) were lymphoid compared with myeloid (n = 8, 14.8%). Of the lymphoid malignancies, 35 (76.1%) were lymphomas with near-equal distribution of Hodgkin’s and non-Hodgkin’s subtypes. The non-Hodgkin’s lymphoma cases included eight diffuse large B cell lymphoma cases, three follicular lymphoma cases, two anaplastic large cell lymphoma cases, four unspecified B cell lymphoma cases, and one mycosis fungoides case. Of the lymphoid cancers, six (13.0%) were multiple myeloma, four (8.7%) were acute lymphoblastic leukemia, and one (2.2%) was chronic lymphocytic leukemia. The myeloid cancer cases included two (25.0%) with acute myeloid leukemia, four (50.0%) chronic myeloid leukemia, and two (25.0%) with myeloproliferative disorder. The myeloproliferative disorder cases included one member diagnosed with polycythemia vera at age 5 and one diagnosed with hypereosinophilic syndrome at age 27.

The characteristics of EYA diagnosed with each of the hematologic cancers are shown in Table 3. Of the 54 hematologic cancer cases, the median age at cancer diagnoses was 35 (range 5–47 years). Chronic myeloid leukemia was diagnosed at a median age of 34 (range 24–39 years), and acute lymphocytic leukemia was diagnosed at a median age of 29 (range 16–45 years). The median age of diagnosis of multiple myeloma was 39 (range 28–47). The median age for non-Hodgkin’s lymphoma was also 39 (ages ranged from 22 to 43). The majority (n = 4, 66.7%) of multiple myeloma cases were reported in females, whereas the majority of chronic myeloid leukemia (n = 3, 75.0%) and chronic lymphocytic leukemia (n = 1, 100%) were reported in males.

Characteristics of EYA diagnosed with the most common types of cancers stratified by sex are presented in Table A1 (female) and Table A2 (male) in Appendix A.

## 4. Discussion

The dust, smoke and fumes from the collapse of the WTC towers resulted in exposures to known and suspected carcinogens, including polycyclic aromatic hydrocarbons, asbestos, benzene, and dioxins [3,13,14,15,16], all of which have been shown to be associated with development of multiple types of cancer. Studies have previously reported cancer rates in recovery and rescue workers exposed to the WTC emissions and cancer characteristics in the WTC EHC population at large [20,22,23,27,29,45]. Here, we describe the distribution of cancer diagnoses in a cohort of EYA “survivors” who self-referred to the WTC EHC and were certified under the WTC Health Program to gain insight in and promote future study of the relationship between environmental exposures and carcinogenesis in EYA. 

In contrast to cancer studies of rescue and recovery workers, the population in this study includes subjects who were less than 20 years old on 9/11 and who were ethnically and racially diverse. Many (>50%) were women. They reported a diverse history of acute and chronic exposure to WTC dust with potential exposure as local workers, residents, students, or commuters. Overall, breast cancer was the most commonly diagnosed cancer, followed by thyroid, then combined hematopoietic and lymphoid cancers. Thyroid, head and neck, and testicular cancers were the most common cancer diagnoses in EYA men, whereas breast cancers, thyroid cancers, and lymphomas were the most common cancer diagnoses in EYA women. 

Many of the cancers documented in this cohort, including myeloma and chronic myeloid leukemia, are relatively rare in the general population and especially in younger age groups. The cases of peritoneal mesothelioma and breast cancers in male EYA are particularly noteworthy, as were cancer diagnoses in the very young, including polycythemia vera at age 5 and thyroid cancer at age 13. Cases of non-Hodgkin’s lymphoma are of interest in this cohort due to the association of this cancer with benzene exposure [46]. 

The Centers for Disease Control and Prevention have linked many types of cancer to risk factors including obesity and tobacco use in the US population. The majority of EYA diagnosed with cancers typically expected to be associated with elevated BMI (colorectal, kidney, thyroid and head and neck cancers [44]) did indeed have an elevated BMI upon evaluation. By contrast, the majority of EYA diagnosed with cancers typically expected to be associated with tobacco use (lung, head and neck, colorectal and kidney cancers [43]) were actually never smokers. 

This study has important strengths. This is the first description of cancers in WTC-exposed local young adult community members compared with previous studies describing cancers reported in older survivor populations and in responders including rescue and recovery workers. This study of the EYA cohort allows for unique insights not afforded by studies of other cohorts exposed to the WTC disaster. Our “survivor” population includes members that were much younger than those of the responder populations on 9/11, a much higher percentage of women, greater ethnic diversity, and a greater diversity of exposures to WTC emissions from collapses of the WTC towers and traumatic psychological stress [20,22,23,25,26,27,29,45]. Inclusion of a majority percentage young adult women in the WTC EHC, and in EYA specifically, allowed us to describe WTC-related cancer characteristics in this otherwise understudied group, to begin to describe differences in cancer characteristics among WTC-exposed women and men, and to offer unique insights for future studies of WTC-related health effects in young women. A limitation of this study is that the WTC EHC is comprised of self-referred members with self-reported risk factors, including WTC emission exposure and tobacco use. The WTC EHC members are subject to selection bias, including potential selection reporting of cancers designated as “certifiable” by the WTCHP. For these reasons, we cannot directly determine cancer incidence or mortality rates in this population. WTC EHC enrollees are offered routine screening within the program, which may explain the detection of some, but not all, cancer diagnoses since many enroll with previous cancer diagnoses and we do not report overall cancer prevalence. Importantly, some patients may have died from these cancers before they were identified for inclusion in this study. In addition, these data cannot be utilized to study cancer characteristics or outcomes from exposure to a specific carcinogenic entity as WTC dust contained a mixture of carcinogens as described.

The description of a diverse variety of solid and lymphoid and hematopoietic tissue malignancies supports the need to further study these cancers. Furthermore, the observation of rare as well as common cancers in this younger population may provide insight regarding environmental exposures and the underlying mechanisms of cancer development. Hence, this study sets the stage for future studies on young adults with environmental exposures, the relationship of environmental exposures to cancer characteristics, and the underlying mechanisms of carcinogenesis. The potential to continually follow these younger patients may provide additional insight into cancer behavior and prognosis. Data from the WTC EHC pan-cancer database allows us to study key carcinogenic mechanisms and processes in future studies by investigating specific cancer pathologic and histologic characteristics, risk factors, and related biomarker information.

## 5. Conclusions

We provide an initial description of cancers in EYA survivors (i.e., age 30 or younger on 9/11) with exposure to the WTC dust, smoke, and fumes. This description provides an overview of the cancers diagnosed in a younger 9/11 civilian cohort with potentially important differences observed between male and female members and with those observed in the general population. This study will facilitate future analyses of this population as well as clinical management. The pathologic and histologic characteristics of cancers with assessment of cancer risk factors and cancer-specific biomarker information in our WTC EHC pan-cancer database will allow for future analyses of individual and group cancer behaviors in this group, as well as long-term follow-up studies of this population. These studies have the potential to provide information on cancer characteristics observed in the WTC EHC in comparison with the general population, insights into the relationship of carcinogenesis to WTC and other environmental exposures, and follow up of early age environmentally exposed people for cancer development.

## Figures and Tables

**Table 1:** Characteristics of EYA with cancer included in the WTC EHC cancer analysis.

	Level	Overall	Age at 9/11 0–10	Age at 9/11 11–20	Age at 9/11 21–30
n		269	6	42	221
Age at 9/11, median [range]		26 [0, 30]	6 [0, 10]	18 [12, 20]	27 [21, 30]
Gender, n (%)	Female	172 (63.9)	2 (33.3)	27 (64.3)	143 (64.7)
	Male	97 (36.1)	4 (66.7)	15 (35.7)	78 (35.3)
Race/Ethnicity, n (%)	NH-White	106 (43.8)	1 (33.3)	11 (29.7)	94 (46.5)
	Hispanic	52 (21.5)	0 (0.0)	13 (35.1)	39 (19.3)
	Asian	42 (17.4)	1 (33.3)	9 (24.3)	32 (15.8)
	NH-Black	38 (15.7)	1 (33.3)	4 (10.8)	33 (16.3)
	Native American/Other	4 (1.7)	0 (0.0)	0 (0.0)	4 (2.0)
BMI, n (%)	Normal weight (<25)	62 (34.3)	1 (100.0)	12 (42.9)	49 (32.2)
	Overweight (25–30)	61 (33.7)	0 (0.0)	10 (35.7)	51 (33.6)
	Obese (≥30)	58 (32.0)	0 (0.0)	6 (21.4)	52 (34.2)
Income, n (%)	≤$30,000/year	64 (26.7)	0 (0.0)	12 (30.8)	52 (26.1)
	>$30,000/year	176 (73.3)	2 (100.0)	27 (69.2)	147 (73.9)
Education, n (%)	High school or less	29 (11.2)	2 (50.0)	4 (9.8)	23 (10.7)
	More than high school	231 (88.8)	2 (50.0)	37 (90.2)	192 (89.3)
Caught in WTC dust cloud, n (%)	No	141 (54.2)	3 (75.0)	24 (58.5)	114 (53.0)
	Yes	119 (45.8)	1 (25.0)	17 (41.5)	101 (47.0)
Exposure category, n (%)	Local worker	141 (54.2)	0 (0.0)	6 (14.6)	135 (62.8)
	Resident	52 (20.0)	3 (75.0)	9 (22.0)	40 (18.6)
	Student	49 (18.8)	1 (25.0)	23 (56.1)	25 (11.6)
	Other	18 (6.9)	0 (0.0)	3 (7.3)	15 (7.0)
Smoking history, n (%)	Never	220 (86.6)	4 (100.0)	40 (97.6)	176 (84.2)
	Former smoker	29 (11.4)	0 (0.0)	0 (0.0)	29 (13.9)
	Current smoker	5 (2.0)	0 (0.0)	1 (2.4)	4 (1.9)
Smoking pack-yr, n (%)	≤5 pack-year	234 (92.1)	4 (100.0)	40 (97.6)	190 (90.9)
	>5 pack-year	20 (7.9)	0 (0.0)	1 (2.4)	19 (9.1)

**Table 2:** Characteristics of EYA diagnosed with the 10 most common solid cancers included in the WTC EHC cancer analysis.

	Level	Breast	Thyroid	Head and Neck Sites	Colon and Rectum	Kidney	Testis	Brain and Spinal Cord	Melanoma of the Skin	Soft Tissue Sarcoma	Lung
n		76	55	15	12	11	11	9	6	5	4
Age at 9/11, median [range]		26 [14, 30]	23 [1, 30]	26 [16, 30]	24 [16, 30]	27 [0, 30]	28 [20, 29]	26 [21, 30]	26 [21, 29]	28 [22, 29]	28 [25, 29]
Age at diagnosis, median [range]		40 [27, 48]	35 [13, 46]	37 [26, 46]	38 [29, 44]	40 [7, 46]	40 [28, 43]	38 [30, 46]	34 [28, 45]	39 [29, 47]	39 [34, 45]
Gender, n (%)	Female	74 (97.4)	42 (76.4)	4 (26.7)	6 (50.0)	3 (27.3)	0 (0.0)	6 (66.7)	4 (66.7)	2 (40.0)	3 (75.0)
	Male	2 (2.6)	13 (23.6)	11 (73.3)	6 (50.0)	8 (72.7)	11 (100.0)	3 (33.3)	2 (33.3)	3 (60.0)	1 (25.0)
Race/Ethnicity, n (%)	NH-White	28 (36.8)	20 (46.5)	5 (33.3)	7 (70.0)	6 (60.0)	6 (60.0)	2 (28.6)	4 (80.0)	2 (40.0)	3 (75.0)
	Hispanic	14 (18.4)	9 (20.9)	1 (6.7)	2 (20.0)	2 (20.0)	3 (30.0)	2 (28.6)	1 (20.0)	0 (0.0)	1 (25.0)
	Asian	15 (19.7)	10 (23.3)	4 (26.7)	0 (0.0)	1 (10.0)	0 (0.0)	0 (0.0)	0 (0.0)	2 (40.0)	0 (0.0)
	NH-Black	17 (22.4)	4 (9.3)	5 (33.3)	1 (10.0)	1 (10.0)	1 (10.0)	2 (28.6)	0 (0.0)	1 (20.0)	0 (0.0)
	Native American/Other	2 (2.6)	0 (0.0)	0 (0.0)	0 (0.0)	0 (0.0)	0 (0.0)	1 (14.3)	0 (0.0)	0 (0.0)	0 (0.0)
BMI, n (%)	Normal weight (<25)	21 (40.4)	12 (36.4)	5 (55.6)	3 (42.9)	3 (30.0)	2 (20.0)	0 (0.0)	0 (0.0)	1 (25.0)	1 (25.0)
	Overweight (25–30)	10 (19.2)	15 (45.5)	1 (11.1)	0 (0.0)	3 (30.0)	4 (40.0)	1 (25.0)	1 (33.3)	2 (50.0)	1 (25.0)
	Obese (≥30)	21 (40.4)	6 (18.2)	3 (33.3)	4 (57.1)	4 (40.0)	4 (40.0)	3 (75.0)	2 (66.7)	1 (25.0)	2 (50.0)
Income, n (%)	≤$30,000/year	15 (21.1)	15 (31.9)	5 (35.7)	2 (20.0)	1 (10.0)	3 (30.0)	4 (50.0)	0 (0.0)	1 (25.0)	1 (25.0)
	>$30,000/year	56 (78.9)	32 (68.1)	9 (64.3)	8 (80.0)	9 (90.0)	7 (70.0)	4 (50.0)	5 (100.0)	3 (75.0)	3 (75.0)
Caught in WTC dust cloud, n (%)	No	43 (57.3)	22 (42.3)	8 (53.3)	8 (72.7)	5 (50.0)	6 (60.0)	4 (50.0)	4 (66.7)	2 (40.0)	3 (75.0)
	Yes	32 (42.7)	30 (57.7)	7 (46.7)	3 (27.3)	5 (50.0)	4 (40.0)	4 (50.0)	2 (33.3)	3 (60.0)	1 (25.0)
Exposure category, n (%)	Local worker	43 (57.3)	22 (42.3)	7 (46.7)	4 (36.4)	7 (70.0)	5 (50.0)	5 (62.5)	3 (50.0)	3 (60.0)	3 (75.0)
	Resident	12 (16.0)	8 (15.4)	6 (40.0)	3 (27.3)	0 (0.0)	2 (20.0)	1 (12.5)	2 (33.3)	1 (20.0)	0 (0.0)
	Student	15 (20.0)	17 (32.7)	2 (13.3)	2 (18.2)	3 (30.0)	2 (20.0)	2 (25.0)	1 (16.7)	0 (0.0)	1 (25.0)
	Other	5 (6.7)	5 (9.6)	0 (0.0)	2 (18.2)	0 (0.0)	1 (10.0)	0 (0.0)	0 (0.0)	1 (20.0)	0 (0.0)
Smoking history, n (%)	Never	66 (88.0)	49 (96.1)	11 (84.6)	8 (80.0)	8 (80.0)	8 (80.0)	6 (85.7)	6 (100.0)	5 (100.0)	1 (25.0)
	Former smoker	9 (12.0)	2 (3.9)	2 (15.4)	2 (20.0)	2 (20.0)	2 (20.0)	1 (14.3)	0 (0.0)	0 (0.0)	3 (75.0)
	Current smoker	0 (0.0)	0 (0.0)	0 (0.0)	0 (0.0)	0 (0.0)	0 (0.0)	0 (0.0)	0 (0.0)	0 (0.0)	0 (0.0)

**Table 3:** Characteristics of EYA diagnosed with hematologic cancers included in the WTC EHC cancer analysis.

	Level	Non-Hodgkin Lymphoma	Hodgkin Lymphoma	Multiple Myeloma	ALL	CML	AML	CLL
n		18	17	6	4	4	2	1
Age at 9/11, median [range]		27 [15, 30]	23 [8, 29]	26 [16, 28]	21 [3, 27]	19 [17, 25]	28 [27, 30]	28 [28, 28]
Age at diagnosis, median [range]		39 [22, 43]	34 [14, 43]	39 [28, 47]	29 [16, 45]	34 [24, 39]	33 [32, 34]	38 [38, 38]
Gender, n (%)	Female	11 (61.1)	8 (47.1)	4 (66.7)	1 (25.0)	1 (25.0)	1 (50.0)	0 (0.0)
	Male	7 (38.9)	9 (52.9)	2 (33.3)	3 (75.0)	3 (75.0)	1 (50.0)	1 (100.0)
Race/Ethnicity, n (%)	NH-White	5 (35.7)	6 (40.0)	1 (16.7)	0 (0.0)	1 (25.0)	1 (50.0)	0 (0.0)
	Hispanic	5 (35.7)	6 (40.0)	2 (33.3)	1 (33.3)	0 (0.0)	1 (50.0)	0 (0.0)
	Asian	2 (14.3)	0 (0.0)	0 (0.0)	2 (66.7)	3 (75.0)	0 (0.0)	1 (100.0)
	NH-Black	2 (14.3)	3 (20.0)	2 (33.3)	0 (0.0)	0 (0.0)	0 (0.0)	0 (0.0)
	Native American/Other	0 (0.0)	0 (0.0)	1 (16.7)	0 (0.0)	0 (0.0)	0 (0.0)	0 (0.0)
BMI, n (%)	Normal weight (<25)	3 (20.0)	5 (62.5)	1 (50.0)	2 (66.7)	1 (50.0)	1 (50.0)	0 (0.0)
	Overweight (25–30)	9 (60.0)	3 (37.5)	1 (50.0)	1 (33.3)	0 (0.0)	1 (50.0)	1 (100.0)
	Obese (≥30)	3 (20.0)	0 (0.0)	0 (0.0)	0 (0.0)	1 (50.0)	0 (0.0)	0 (0.0)
Income, n (%)	≤$30,000/year	5 (33.3)	2 (13.3)	2 (40.0)	2 (66.7)	1 (25.0)	0 (0.0)	0 (0.0)
	>$30,000/year	10 (66.7)	13 (86.7)	3 (60.0)	1 (33.3)	3 (75.0)	1 (100.0)	1 (100.0)
Caught in WTC dust cloud, n (%)	No	12 (66.7)	8 (50.0)	3 (60.0)	4 (100.0)	3 (75.0)	1 (50.0)	1 (100.0)
	Yes	6 (33.3)	8 (50.0)	2 (40.0)	0 (0.0)	1 (25.0)	1 (50.0)	0 (0.0)
Exposure category, n (%)	Local worker	13 (72.2)	9 (56.2)	3 (60.0)	1 (25.0)	0 (0.0)	1 (50.0)	1 (100.0)
	Resident	2 (11.1)	5 (31.2)	0 (0.0)	2 (50.0)	2 (50.0)	0 (0.0)	0 (0.0)
	Student	1 (5.6)	2 (12.5)	1 (20.0)	1 (25.0)	2 (50.0)	0 (0.0)	0 (0.0)
	Other	2 (11.1)	0 (0.0)	1 (20.0)	0 ( 0.0)	0 (0.0)	1 (50.0)	0 (0.0)
Smoking history, n (%)	Never	16 (88.9)	15 (93.8)	4 (80.0)	3 (75.0)	3 (75.0)	2 (100.0)	1 (100.0)
	Former smoker	1 (5.6)	1 (6.2)	0 (0.0)	1 (25.0)	1 (25.0)	0 (0.0)	0 (0.0)
	Current smoker	1 (5.6)	0 (0.0)	1 (20.0)	0 (0.0)	0 (0.0)	0 (0.0)	0 (0.0)

## Data Availability

The datasets analyzed here in the WTC EHC Data Center are not publicly available but de-identified and anonymized information are potentially available upon request.

## Glossary

**Acronym**	**Definition**
BMI	Body mass index
CDC	Centers for Disease Control and Prevention
EYA	World Trade Center-exposed survivors who were exposed at a young age, age 30 or younger on 11 September 2001
NIOSH	National Institute for Occupational Safety & Health
NYS	New York State
WTC	World Trade Center
WTC EHC	World Trade Center Environmental Health Center
WTCHP	World Trade Center Health Program
WTCHR	World Trade Center Health Registry
WTC EHC PCDB	World Trade Center Environmental Health Center pan-cancer database
9/11	11 September 2001

## Appendix A

**Table A1:** Characteristics of female EYA diagnosed with the most common cancers included in the WTC EHC cancer analysis (as of 31 July 2021).

	Level	Breast	Thyroid	Brain and Spinal Cord	Colon and Rectum	Head and Neck Sites	Melanoma of the Skin	Kidney	Lung	Pancreas	Soft Tissue Sarcoma	Non-Hodgkin Lymphoma	Hodgkin Lymphoma	Myeloma	Leukemia
n		74	42	6	6	4	4	3	3	2	2	11	8	4	3
Age at 9/11, median [range]		26 [14, 30]	23 [1, 30]	28 [23, 30]	26 [19, 30]	21 [16, 22]	26 [21, 29]	28 [18, 30]	28 [28, 29]	29 [29, 29]	26 [23, 29]	27 [15, 30]	26 [13, 29]	28 [25, 28]	27 [20, 30]
Age at diagnosis, median [range]		40 [27, 48]	35 [13, 46]	40 [35, 46]	41 [29, 44]	35 [26, 35]	34 [29, 42]	45 [32, 46]	40 [34, 45]	42 [37, 47]	43 [39, 47]	41 [22, 43]	32 [19, 39]	42 [38, 47]	35 [34, 45]
Race/Ethnicity, n (%)	NH-White	27 (36.5)	16 (47.1)	2 (40.0)	4 (80.0)	0 (0.0)	3 (75.0)	1 (33.3)	2 (66.7)	1 (50.0)	0 (0.0)	2 (25.0)	2 (28.6)	0 (0.0)	1 (33.3)
	Hispanic	14 (18.9)	9 (26.5)	1 (20.0)	1 (20.0)	1 (25.0)	1 (25.0)	2 (66.7)	1 (33.3)	1 (50.0)	0 (0.0)	3 (37.5)	4 (57.1)	2 (50.0)	1 (33.3)
	Asian	15 (20.3)	7 (20.6)	0 (0.0)	0 (0.0)	2 (50.0)	0 (0.0)	0 (0.0)	0 (0.0)	0 (0.0)	1 (50.0)	2 (25.0)	0 (0.0)	0 (0.0)	1 (33.3)
	NH-Black	16 (21.6)	2 (5.9)	1 (20.0)	0 (0.0)	1 (25.0)	0 (0.0)	0 (0.0)	0 (0.0)	0 (0.0)	1 (50.0)	1 (12.5)	1 (14.3)	1 (25.0)	0 (0.0)
	Native American/Other	2 (2.7)	0 (0.0)	1 (20.0)	0 (0.0)	0 (0.0)	0 (0.0)	0 (0.0)	0 (0.0)	0 (0.0)	0 (0.0)	0 (0.0)	0 (0.0)	1 (25.0)	0 (0.0)
BMI, n (%)	Normal weight (<25)	21 (41.2)	10 (41.7)	0 (0.0)	3 (100.0)	1 (100.0)	0 (0.0)	0 (0.0)	1 (33.3)	0 (0.0)	0 (0.0)	3 (33.3)	4 (80.0)	0 (NaN)	0 (0.0)
	Overweight (25–30)	10 (19.6)	9 (37.5)	1 (33.3)	0 (0.0)	0 (0.0)	1 (33.3)	1 (33.3)	1 (33.3)	1 (50.0)	1 (50.0)	5 (55.6)	1 (20.0)	0 (NaN)	1 (100.0)
	Obese (≥30)	20 (39.2)	5 (20.8)	2 (66.7)	0 (0.0)	0 (0.0)	2 (66.7)	2 (66.7)	1 (33.3)	1 (50.0)	1 (50.0)	1 (11.1)	0 (0.0)	0 (NaN)	0 (0.0)
Income, n (%)	≤$30,000/year	15 (21.7)	11 (28.9)	3 (60.0)	1 (25.0)	3 (75.0)	0 (0.0)	1 (33.3)	1 (33.3)	0 (0.0)	0 (0.0)	3 (33.3)	1 (12.5)	1 (33.3)	0 (0.0)
	>$30,000/year	54 (78.3)	27 (71.1)	2 (40.0)	3 (75.0)	1 (25.0)	4 (100.0)	2 (66.7)	2 (66.7)	2 (100.0)	2 (100.0)	6 (66.7)	7 (87.5)	2 (66.7)	3 (100.0)
Caught in WTC dust cloud, n (%)	No	41 (56.2)	18 (45.0)	3 (60.0)	3 (60.0)	3 (75.0)	3 (75.0)	1 (33.3)	2 (66.7)	2 (100.0)	1 (50.0)	8 (72.7)	3 (37.5)	2 (66.7)	3 (100.0)
	Yes	32 (43.8)	22 (55.0)	2 (40.0)	2 (40.0)	1 (25.0)	1 (25.0)	2 (66.7)	1 (33.3)	0 (0.0)	1 (50.0)	3 (27.3)	5 (62.5)	1 (33.3)	0 (0.0)
Exposure category, n (%)	Local Worker	42 (57.5)	17 (42.5)	4 (80.0)	2 (40.0)	1 (25.0)	2 (50.0)	2 (66.7)	2 (66.7)	2 (100.0)	2 (100.0)	6 (54.5)	3 (37.5)	2 (66.7)	1 (33.3)
	Resident	11 (15.1)	6 (15.0)	0 (0.0)	1 (20.0)	3 (75.0)	1 (25.0)	0 (0.0)	0 (0.0)	0 (0.0)	0 (0.0)	2 (18.2)	3 (37.5)	0 (0.0)	1 (33.3)
	Student	15 (20.5)	14 (35.0)	1 (20.0)	1 (20.0)	0 (0.0)	1 (25.0)	1 (33.3)	1 (33.3)	0 (0.0)	0 (0.0)	1 (9.1)	2 (25.0)	0 (0.0)	1 (33.3)
	Other	5 (6.8)	3 (7.5)	0 (0.0)	1 (20.0)	0 (0.0)	0 (0.0)	0 (0.0)	0 (0.0)	0 (0.0)	0 (0.0)	2 (18.2)	0 (0.0)	1 (33.3)	0 (0.0)
Smoking history, n (%)	Never	64 (87.7)	38 (97.4)	4 (100.0)	2 (50.0)	4 (100.0)	4 (100.0)	2 (66.7)	1 (33.3)	1 (100.0)	2 (100.0)	11 (100.0)	8 (100.0)	2 (66.7)	2 (66.7)
	Former smoker	9 (12.3)	1 (2.6)	0 (0.0)	2 (50.0)	0 (0.0)	0 (0.0)	1 (33.3)	2 (66.7)	0 (0.0)	0 (0.0)	0 (0.0)	0 (0.0)	0 (0.0)	1 (33.3)
	Current smoker	0 (0.0)	0 (0.0)	0 (0.0)	0 (0.0)	0 (0.0)	0 (0.0)	0 (0.0)	0 (0.0)	0 (0.0)	0 (0.0)	0 (0.0)	0 (0.0)	1 (33.3)	0 (0.0)

**Table A2:** Characteristics of male EYA diagnosed with the most common cancers included in the WTC EHC cancer analysis (as of 31 July 2021).

	Level	Thyroid	Head and Neck Sites	Testis	Kidney	Colon and Rectum	Brain and Spinal Cord	Prostate	Soft Tissue Sarcoma	Breast	Melanoma of the Skin	Small Intestine	Urinary Bladder	Hodgkin Lymphoma	Leukemia	Non-Hodgkin Lymphoma	Myeloma	Myeloproliferative Neoplasm
n		13	11	11	8	6	3	3	3	2	2	2	2	9	8	7	2	2
Age at 9/11, median [range]		26 [18, 30]	27 [20, 30]	28 [20, 29]	26 [0, 28]	24 [16, 27]	25 [21, 26]	27 [24, 29]	28 [22, 29]	24 [23, 25]	26 [24, 27]	26 [23, 29]	26 [25, 28]	21 [8, 28]	21 [3, 28]	29 [20, 30]	18 [16, 20]	22 [19, 26]
Age at diagnosis, median [range]		37 [26, 45]	39 [28, 46]	40 [28, 43]	40 [7, 43]	36 [31, 43]	36 [30, 38]	45 [40, 47]	34 [29, 41]	40 [39, 41]	36 [28, 45]	37 [36, 38]	36 [34, 39]	35 [14, 43]	32 [16, 39]	38 [26, 41]	32 [28, 35]	16 [5, 27]
Race/Ethnicity, n (%)	NH-White	4 (44.4)	5 (45.5)	6 (60.0)	5 (71.4)	3 (60.0)	0 (0.0)	3 (100.0)	2 (66.7)	1 (50.0)	1 (100.0)	1 (50.0)	0 (0.0)	4 (50.0)	1 (14.3)	3 (50.0)	1 (50.0)	2 (100.0)
	Hispanic	0 (0.0)	0 (0.0)	3 (30.0)	0 (0.0)	1 (20.0)	1 (50.0)	0 (0.0)	0 (0.0)	0 (0.0)	0 (0.0)	0 (0.0)	2 (100.0)	2 (25.0)	1 (14.3)	2 (33.3)	0 (0.0)	0 (0.0)
	Asian	3 (33.3)	2 (18.2)	0 (0.0)	1 (14.3)	0 (0.0)	0 (0.0)	0 (0.0)	1 (33.3)	0 (0.0)	0 (0.0)	0 (0.0)	0 (0.0)	0 (0.0)	5 (71.4)	0 (0.0)	0 (0.0)	0 (0.0)
	NH-Black	2 (22.2)	4 (36.4)	1 (10.0)	1 (14.3)	1 (20.0)	1 (50.0)	0 (0.0)	0 (0.0)	1 (50.0)	0 (0.0)	1 (50.0)	0 (0.0)	2 (25.0)	0 (0.0)	1 (16.7)	1 (50.0)	0 (0.0)
	Native American/Other	0 (0.0)	0 (0.0)	0 (0.0)	0 (0.0)	0 (0.0)	0 (0.0)	0 (0.0)	0 (0.0)	0 (0.0)	0 (0.0)	0 (0.0)	0 (0.0)	0 (0.0)	0 (0.0)	0 (0.0)	0 (0.0)	0 (0.0)
BMI, n (%)	Normal weight (<25)	2 (22.2)	4 (50.0)	2 (20.0)	3 (42.9)	0 (0.0)	0 (0.0)	0 (0.0)	1 (50.0)	0 (0.0)	0 (NaN)	0 (0.0)	0 (0.0)	1 (33.3)	4 (57.1)	0 (0.0)	1 (50.0)	0 (0.0)
	Overweight (25–30)	6 (66.7)	1 (12.5)	4 (40.0)	2 (28.6)	0 (0.0)	0 (0.0)	2 (66.7)	1 (50.0)	0 (0.0)	0 (NaN)	0 (0.0)	0 (0.0)	2 (66.7)	2 (28.6)	4 (66.7)	1 (50.0)	2 (100.0)
	Obese (≥30)	1 (11.1)	3 (37.5)	4 (40.0)	2 (28.6)	4 (100.0)	1 (100.0)	1 (33.3)	0 (0.0)	1 (100.0)	0 (NaN)	1 (100.0)	2 (100.0)	0 (0.0)	1 (14.3)	2 (33.3)	0 (0.0)	0 (0.0)
Income, n (%)	≤$30,000/year	4 (44.4)	2 (20.0)	3 (30.0)	0 (0.0)	1 (16.7)	1 (33.3)	0 (0.0)	1 (50.0)	0 (0.0)	0 (0.0)	1 (50.0)	1 (50.0)	1 (14.3)	3 (50.0)	2 (33.3)	1 (50.0)	1 (50.0)
	>$30,000/year	5 (55.6)	8 (80.0)	7 (70.0)	7 (100.0)	5 (83.3)	2 (66.7)	3 (100.0)	1 (50.0)	2 (100.0)	1 (100.0)	1 (50.0)	1 (50.0)	6 (85.7)	3 (50.0)	4 (66.7)	1 (50.0)	1 (50.0)
Caught in WTC dust cloud, n (%)	No	4 (33.3)	5 (45.5)	6 (60.0)	4 (57.1)	5 (83.3)	1 (33.3)	1 (33.3)	1 (33.3)	2 (100.0)	1 (50.0)	0 (0.0)	0 (0.0)	5 (62.5)	6 (75.0)	4 (57.1)	1 (50.0)	1 (50.0)
	Yes	8 (66.7)	6 (54.5)	4 (40.0)	3 (42.9)	1 (16.7)	2 (66.7)	2 (66.7)	2 (66.7)	0 (0.0)	1 (50.0)	2 (100.0)	2 (100.0)	3 (37.5)	2 (25.0)	3 (42.9)	1 (50.0)	1 (50.0)
Exposure category, n (%)	Local Worker	5 (41.7)	6 (54.5)	5 (50.0)	5 (71.4)	2 (33.3)	1 (33.3)	2 (66.7)	1 (33.3)	1 (50.0)	1 (50.0)	2 (100.0)	2 (100.0)	6 (75.0)	2 (25.0)	7 (100.0)	1 (50.0)	1 (50.0)
	Resident	2 (16.7)	3 (27.3)	2 (20.0)	0 (0.0)	2 (33.3)	1 (33.3)	1 (33.3)	1 (33.3)	1 (50.0)	1 (50.0)	0 (0.0)	0 (0.0)	2 (25.0)	3 (37.5)	0 (0.0)	0 (0.0)	0 (0.0)
	Student	3 (25.0)	2 (18.2)	2 (20.0)	2 (28.6)	1 (16.7)	1 (33.3)	0 (0.0)	0 (0.0)	0 (0.0)	0 (0.0)	0 (0.0)	0 (0.0)	0 (0.0)	2 (25.0)	0 (0.0)	1 (50.0)	1 (50.0)
	Other	2 (16.7)	0 (0.0)	1 (10.0)	0 (0.0)	1 (16.7)	0 (0.0)	0 (0.0)	1 (33.3)	0 (0.0)	0 (0.0)	0 (0.0)	0 (0.0)	0 (0.0)	1 (12.5)	0 (0.0)	0 (0.0)	0 (0.0)
Smoking history, n (%)	Never	11 (91.7)	7 (77.8)	8 (80.0)	6 (85.7)	6 (100.0)	2 (66.7)	2 (66.7)	3 (100.0)	2 (100.0)	2 (100.0)	2 (100.0)	1 (50.0)	7 (87.5)	7 (87.5)	5 (71.4)	2 (100.0)	0 (0.0)
	Former smoker	1 (8.3)	2 (22.2)	2 (20.0)	1 (14.3)	0 (0.0)	1 (33.3)	1 (33.3)	0 (0.0)	0 (0.0)	0 (0.0)	0 (0.0)	1 (50.0)	1 (12.5)	1 (12.5)	1 (14.3)	0 (0.0)	0 (0.0)
	Current smoker	0 (0.0)	0 (0.0)	0 (0.0)	0 (0.0)	0 (0.0)	0 (0.0)	0 (0.0)	0 (0.0)	0 (0.0)	0 (0.0)	0 (0.0)	0 (0.0)	0 (0.0)	0 (0.0)	1 (14.3)	0 (0.0)	1 (100.0)
